# Gene expression and characterization of clonally derived murine embryonic brown and brite adipocytes

**DOI:** 10.1002/2211-5463.13861

**Published:** 2024-07-07

**Authors:** Cristina Velez‐delValle, Claudia Patricia Hernandez‐Mosqueira, Lidia Itzel Castro‐Rodriguez, Alfredo Vazquez‐Sandoval, Meytha Marsch‐Moreno, Walid Kuri‐Harcuch

**Affiliations:** ^1^ Department of Cell Biology Center for Research and Advanced Studies (Cinvestav) Mexico City Mexico

**Keywords:** adipocytes, adipogenesis, brite/beige, brown, differentiation, gene expression

## Abstract

White adipocytes store energy, while brown and brite adipocytes release heat via nonshivering thermogenesis. In this study, we characterized two murine embryonic clonal preadipocyte lines, EB5 and EB7, each displaying unique gene marker expression profiles. EB5 cells differentiate into brown adipocytes, whereas EB7 cells into brite (also known as beige) adipocytes. To draw a comprehensive comparison, we contrasted the gene expression patterns, adipogenic capacity, as well as carbohydrate and lipid metabolism of these cells to that of F442A, a well‐known white preadipocyte and adipocyte model. We found that commitment to differentiation in both EB5 and EB7 cells can be induced by 3‐Isobutyl‐1‐methylxanthine/dexamethasone (Mix/Dex) and staurosporine/dexamethasone (St/Dex) treatments. Additionally, the administration of rosiglitazone significantly enhances the brown and brite adipocyte phenotypes. Our data also reveal the involvement of a series of genes in the transcriptional cascade guiding adipogenesis, pinpointing GSK3β as a critical regulator for both EB5 and EB7 adipogenesis. In a developmental context, we observe that, akin to brown fat progenitors, brite fat progenitors make their appearance in murine development by 11–12 days of gestation or potentially earlier. This result contributes to our understanding of adipocyte lineage specification during embryonic development. In conclusion, EB5 and EB7 cell lines are valuable for research into adipocyte biology, providing insights into the differentiation and development of brown and beige adipocytes. Furthermore, they could be useful for the characterization of drugs targeting energy balance for the treatment of obesity and metabolic diseases.

## Graphical abstract

Murine brown and beige fat progenitors arise by 11–12 days of embryonic life. We studied gene expression profiles of EB5 (brown) and EB7 (beige), two murine embryonic clonal preadipocyte lines. These cells were compared for adipose differentiation, cold response, carbohydrate, lipid metabolism, and thermogenic gene expression. GSK3β is key in regulating brown and beige adipogenesis.

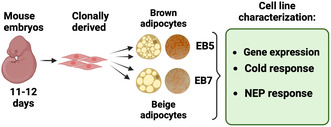

**Abbreviations**
ACC2: acetyl‐CoA carboxylase 2
Atgl: adipose triglyceride lipase
BAT: brown adipose tissue
BRT: “brown in white” adipose tissue
Cd81: cluster of differentiation 81
CEB/Pα: CCAAT/enhancer binding protein alpha
CEB/Pβ: CCAAT/enhancer binding protein beta
ChREBP: carbohydrate response element binding protein
CIDE‐A: cell death activator
Cpt1b: palmitoyl‐CoA transferase‐1b
Dex: dexamethasone
DMEM: Dulbecco's Modified Eagle's Medium
FABP4: fatty acid binding protein 4
Fasn: fatty acid synthase
GLUT4: glucose transporter 4
GPD1: glycerophosphate dehydrogenase
GSK3β: glycogen synthase kinase‐3 beta
HSL: hormone sensitive lipase
Lhx8: LIM homeobox protein 8
LPL: lipoprotein lipase
Mix: 3‐isobutyl‐1‐methylxanthine
MUFA: mono unsaturated fatty acids
Myf‐5: myogenic factor 5
NAd: nonadipogenic medium
PDGFRα: platelet‐derived growth factor receptor alpha
Pparg2: peroxisome proliferator activated receptor gamma 2
Ppargc1α: peroxisome proliferative activated receptor gamma coactivator 1 alpha
PRDM16: PR domain zinc finger protein 16
RMD: 1 μm rosiglitazone, 0.5 mm mix, and 1 μm Dex
Rplp0: ribosomal phosphoprotein large P0
Sca1: spinocerebellar ataxia type 1
Scd1: stearoyl‐CoA desaturase‐1
SDR: St/Dex/Ros
SREBP1c: sterol regulatory element‐binding protein 1 isoform c
St: staurosporine
Tcf21: transcription factor 21
Tmem26: transmembrane protein 26
UCP1: uncoupling protein 1
WAT: white adipose tissue
Zic1: zinc finger protein of the cerebellum 1
β_3_‐Ars: β_3_‐adrenergic receptors

Adipose tissues are highly dynamic organs that play a central role in metabolic and energy homeostasis. White, brown, and brite (beige) adipocytes have important metabolic properties that contribute to whole‐body metabolic regulation, reviewed in Scheele and Wolfrum [1].

In rodents, brown adipocytes are pronounced in the interscapular fat depots and are derived from a cell lineage characterized by *Myf‐5* ^+^ (Myogenic factor 5) skeletal muscle‐like cells [2]. Beige or brite adipocytes have brown adipocyte activity and are found in predominantly white adipose tissue (WAT) and near the blood vessels. Their cell progenitors reside within the vasculature among the mural cells are not derived from the *Myf‐5* ^+^ lineage [3], thus making them genetically distinct from brown fat. Brown adipose tissue (BAT) and “brown in white” adipose tissue (BRT) are thought to be suitable targets for obesity therapy because of their ability to regulate energy expenditure. Excess fats can be broken down by lipid oxidation and provide fuel for thermogenesis in mitochondria of brown and brite adipocytes. The fatty acids released into the circulation from WAT and those from the brown and brite adipocytes lipid stores are fuel sources for thermogenesis. BAT burns excess lipids through obligatory and nonshivering thermogenesis, while BRT burns excess lipids through nonshivering thermogenesis in response to chronic cold exposure and by β_3_‐adrenergic stimuli; some estimates indicate that in the human body brite are more abundant than brown adipocytes [4, 5, 6, 7, 8].

Brown and brite adipocytes carry out thermogenesis through Uncoupling protein 1 (UCP1), the uncoupling respiratory protein at the mitochondrial inner membrane. Basal UCP1 expression is higher in brown fat cells than in brite, and lowest in white adipocytes [9]. Brown‐ and brite‐mediated energy expenditure can improve glucose and lipid homeostasis through increased fatty acid beta‐oxidation and thermogenesis [10, 11, 12, 13, reviewed by 14]. This raises the possibility to treat obesity through increasing mass and/or stimulating the activity of BAT and/or BRT (i.e., browning or britening) [8, 15]. The observation that BAT mass or/and activity is reduced during aging and in subjects with obesity and diabetes [16], it also suggests that increasing BAT and BRT mass or activity might lead to promising therapies for those diseases.

Several proteins regulate adipogenesis of brown and brite fat cell progenitors. PR Domain Zinc Finger Protein 16 (PRDM16), a transcriptional zinc‐finger histone methyltransferase expressed in brown and beige adipocytes, as well as in smooth muscle cells and a few other tissues, controls the thermogenic program in brown and brite adipocytes [11]. In mouse embryos of 9–12 days gestation, PRDM16 controls the muscle‐brown fat differentiation decision in the *Myf‐5* ^+^ progenitors, and its expression leads to brown adipogenesis [2, 17, 18]. In the mouse embryo brown fat arises at 9–12 days of gestation, whereas subcutaneous WAT arises at 14–18 days [19]. Adult WAT has brite fat progenitors likely derived from *Myf5*‐precursors expressing platelet‐derived growth factor receptor alpha (PDGFRα^+^) [8, 19, 20, 21], and a subset of progenitors marked by PDGFRα, Spinocerebellar ataxia type 1 (*Sca1*), and Cluster of Differentiation 81 (*Cd81*) [22]. While it is well established that brown and white adipocyte progenitors originate during embryonic life, the developmental timing of brite progenitors remains unclear, whether they emerge during embryonic stages or postnatally.

Brown and brite adipocyte models have been investigated in humans, animals, virally immortalized cell lines, and in primary cell cultures from BAT and from the stromal‐vascular fraction of adipose tissue [23, 24, 25]. Brite adipose cell culture models are less common than those of brown adipose, which limit research into the molecular regulation of BRT differentiation and function. Despite that PRDM16 is expressed in mouse embryos, limited studies of brown or brite adipogenesis have been reported in embryonic cellular models [2, 17, 18]. While the C3H10T1/2 cell line was established from 14‐ to 17‐day‐old mouse embryos [26] and has been used to study brown adipogenesis [27, 28, 29] and expression of select beige markers [30, 31, 32, 33, 34], detailed genetic and cold‐response analysis related to the beige phenotype is lacking.

Research with cell cultures can aid in a better understanding of the mechanisms that regulate brown and brite cell differentiation and metabolism, which, in turn, may lead to new treatments for metabolic diseases. Thus, to better understand adipogenesis and early development of brown and brite adipocytes, we established cell culture models of these cells using previously derived cells from 11‐ to 12‐day murine embryos [35]. Following the derivation protocol used for the white 3T3‐F442A (F442A) preadipocytes [36, 37], we derived two adipogenic clonal cell lines EB5 and EB7 from mouse strain NMRI embryos.

de Jong *et al*. [38] described experiments using adipose tissues from brown (interscapular), brite (inguinal), and white (epididymal) regions of NMRI mice, along with primary cell cultures derived from these tissues, to assess various genetic markers. Most of these markers (*Car4*, *Cited1*, *Ebf3*, *Eva1*, *Fbxo31*, *Fgf21*, *Lhx8*, *Hoxc8*, and *Hoxc9*) were not strongly indicative of tissue type. However, markers such as *Zic1* (specific to brown adipose tissue), *Cd137*, *Epsti1*, *Tbx1*, *Tmem26* (associated with brite adipose tissue), and *Tcf21* (indicative of white adipose tissue) were informative for distinguishing between these tissues. Notably, *Lhx8* expression was significantly higher in interscapular BAT compared to inguinal BRT and was undetectable in epididymal WAT. *Lhx8* is found in brown and only seen in brite when this tissue is activated with β‐adrenergic agonists [38, 39]. The study also acknowledges discrepancies in marker reliability reported in the literature, suggesting that variations could be due to factors such as mouse strain differences (C57BL/6 versus NMRI), cell culture conditions, among others. Consequently, we used a subset of these genetic markers previously reported for the NMRI strain [38] to evaluate the EB5 and EB7 clones, which originated from NMRI mice.

We examined the expression profiles of the genetic markers: Zinc finger protein of the cerebellum 1 (*Zic1*) and LIM homeobox protein 8 (*Lhx8*) for brown, Transmembrane protein 26 (*Tmem26*) for brown and brite, and Transcription factor 21 (*Tcf21*) for white. The limitations of genetic marker analysis in providing a definitive identification of adipocyte lineage, as noted by de Jong *et al*., led us to complement our study with an assessment of the clones' response to cold exposure [40]. Integrating the genetic and the response to cold data enabled us to identify the lineage of the EB5 and EB7 clones, each with the ability to differentiate into adipocytes under appropriate culture conditions.

The expression of carbohydrate and lipid metabolic genes in brown and brite cultured adipocytes is not well known. Carbohydrate, lipogenic, and lipolytic pathways are important in providing fatty acids into the mitochondria for thermogenesis in BAT and BRT. Glucose might be available through the GLUT4 transporter, while the fatty acids are available through the following metabolic pathways: (a) Fatty acid uptake from the bloodstream via triglyceride degradation into fatty acids and glycerol by lipoprotein lipase and lipolysis from the intracytoplasmic triglycerides. (b) Lipogenesis, or the *de novo* fatty acids synthesis and triglycerides. (c) Intracytoplasmic fatty acid transport. (d) Mono‐unsaturated fatty acid synthesis. To get a better understanding of these metabolic pathways in brown and brite adipocytes, we determined the expression levels of select genes that encode crucial enzymes within these pathways in EB5, EB7, and F442A adipocytes: For fatty acid uptake, the genes encoding lipoprotein lipase (LPL, gene *Lpl*) and the intracellular lipolytic proteins, adipose triglyceride lipase (Atgl, gene *Atgl*), and hormone‐sensitive lipase (Lipe, gene *Lipe*). For lipogenic enzymes, fatty acid synthase (Fasn, gene *Fasn*), glycerophosphate dehydrogenase (GPD1, gene *Gpd1*), and *Mlxipl*, the gene encoding for carbohydrate response element binding protein (ChREBP), a helix–loop–helix leucine zipper transcription factor. For transporters, the genes encoding the glucose transporter (GLUT4, gene *Slc2a4*), the intracytoplasmic fatty acid transporters (FABP4, gene *Fapb4*), and CD36 (gene *Cd36*). For beta‐oxidation, *Cpt1b* that encodes the enzyme palmitoyl‐CoA transferase‐1b, a fatty acid transporter into the mitochondria that is in the outer mitochondrial membrane and essential for thermogenesis. An important regulator of CPT1b activity is malonyl‐CoA Acetyl‐CoA Carboxylase 2 (ACC2, gene *Acacb*) catalyzes the formation of malonyl‐CoA, an inhibitor of CPT1b activity. For mono‐unsaturated fatty acid synthesis, the rate‐limiting enzyme, stearoyl‐CoA desaturase‐1 (Scd1, gene *Scd1*) that catalyzes the biosynthesis of Mono Unsaturated Fatty Acids (MUFA), palmitoleyl‐CoA, and oleyl‐CoA from palmitoyl‐CoA and stearoyl‐CoA, respectively. MUFA are key components of triglycerides and membrane phospholipids, as well as precursors for fat oxidation and thermogenesis. For adipokines, adiponectin (*Adipoq*) and leptin (*Lep*).

The β_3_‐adrenergic response indicates brown adipocytes physiological capabilities. Upon rodents' exposure to cold, the sympathetic nervous system releases norepinephrine, a β_3_‐ adrenergic agent, and activates nonshivering thermogenesis in the brown adipocytes through β_3_‐Adrenergic receptors (β_3_‐ARs) [41].

Basal respiration is low in brown adipocytes, but norepinephrine stimulation of β_3_‐ARs in brown adipocytes increased adenylyl cyclase activity, lipolysis, and oxygen consumption (thermogenesis) through increased UCP1 transcript and activity [41, 42, 43, 44, 45, 46, 47]. Silencing β_3_‐ARs reduced all of them and the expression of genes involved in fatty acid metabolism, mitochondrial mass, and thermogenesis, as well [48]. Lipolysis is crucial for UCP1 activation. Atgl and HSL lipases inhibition completely blocked the adrenergic thermogenic response [49]. UCP1 knockdown adipocytes did not initiate thermogenesis in response to β_3_‐adrenergic receptor agonists, but still showed norepinephrine‐induced lipolysis, indicating that UCP1 is the sole mediator of adrenergic‐induced thermogenesis [49]. Activation of β_3_‐adrenergic receptors lead to lipolysis, increased *Ucp1* expression, uncoupled respiration, and fatty acid metabolism.

The response of brown adipocytes to norepinephrine provides insight into their metabolic and functional states beyond the scope of metabolic gene expression. We assessed lipolysis and UCP1 content in EB5 and EB7 adipocytes following norepinephrine stimulation to evaluate their metabolic and functional competence.

Taken together, our findings highlight the potential to map differentiation, disease, and drug pathways in a rapid and robust manner by doing comparative studies with the three types of white, brown, and brite preadipocytes and adipocytes in culture.

## Methods

### Materials

We obtained Dulbecco's Modified Eagle's Medium (DMEM) and TRIzol® reagent from Thermo Fisher Scientific (Waltham, MA, USA). Both calf and adult bovine serum were sourced from HyClone (Logan, UT, USA), a subsidiary of Thermo Fisher Scientific. Adult cat serum was collected from domestic adult cats in strict compliance with the guidelines set forth in the Guide for the Care and Use of Laboratory Animals of the National Institutes of Health. All animal handling procedures were conducted in accordance with protocols approved by the Internal Committee for the Care and Use of Laboratory Animals (CICUAL) at of the Center for Advanced Research (CINVESTAV‐IPN). Epidermal growth factor was procured from Upstate Inc. (Charlottesville, VA, USA). Additional reagents such as insulin, d‐biotin, human transferrin, triiodothyronine, dexamethasone, 3‐Isobutyl‐1methylxanthine, rosiglitazone, staurosporine, SB415286, and Oil Red O were obtained from Sigma‐Aldrich. (Saint Louis, MO, USA). All other chemicals used in the experiments were of analytical grade.

### Cell culture and isolation of the EB5 and EB7 preadipocytes

We previously established a parental cell line from 11 to 12 days NMRI mouse embryos through a regimen of subculture transfers, adhering to protocols previously used for 3T3 cells derived from Swiss mouse embryos [50]. Due to the age of the mouse embryos, we hypothesized that this line could contain brown adipocyte progenitor cells. To investigate this, two successive cloning steps were carried out. Initially, the parental cells were cultured in DMEM supplemented with 10% calf serum. These growing cultures were then trypsinized and seeded at 100 cells per dish into two 100‐mm tissue culture dishes. This led to the derivation of adipogenic clonal cells EB5 and EB7, following methodologies established for white F442A preadipocytes [36, 37]. Once colonies achieved a small yet confluent center, they were subjected to 48 h treatment with an RMD medium. This medium consisted of 1 μm Rosiglitazone, 0.5 mm 3‐Isobutyl‐1‐methylxanthine, and 1 μm dexamethasone (RMD), and was further supplemented with 5 μg·mL^−1^ insulin, 1 μm d‐biotin, and 2 nm triiodothyronine.

**Fig. 1:**
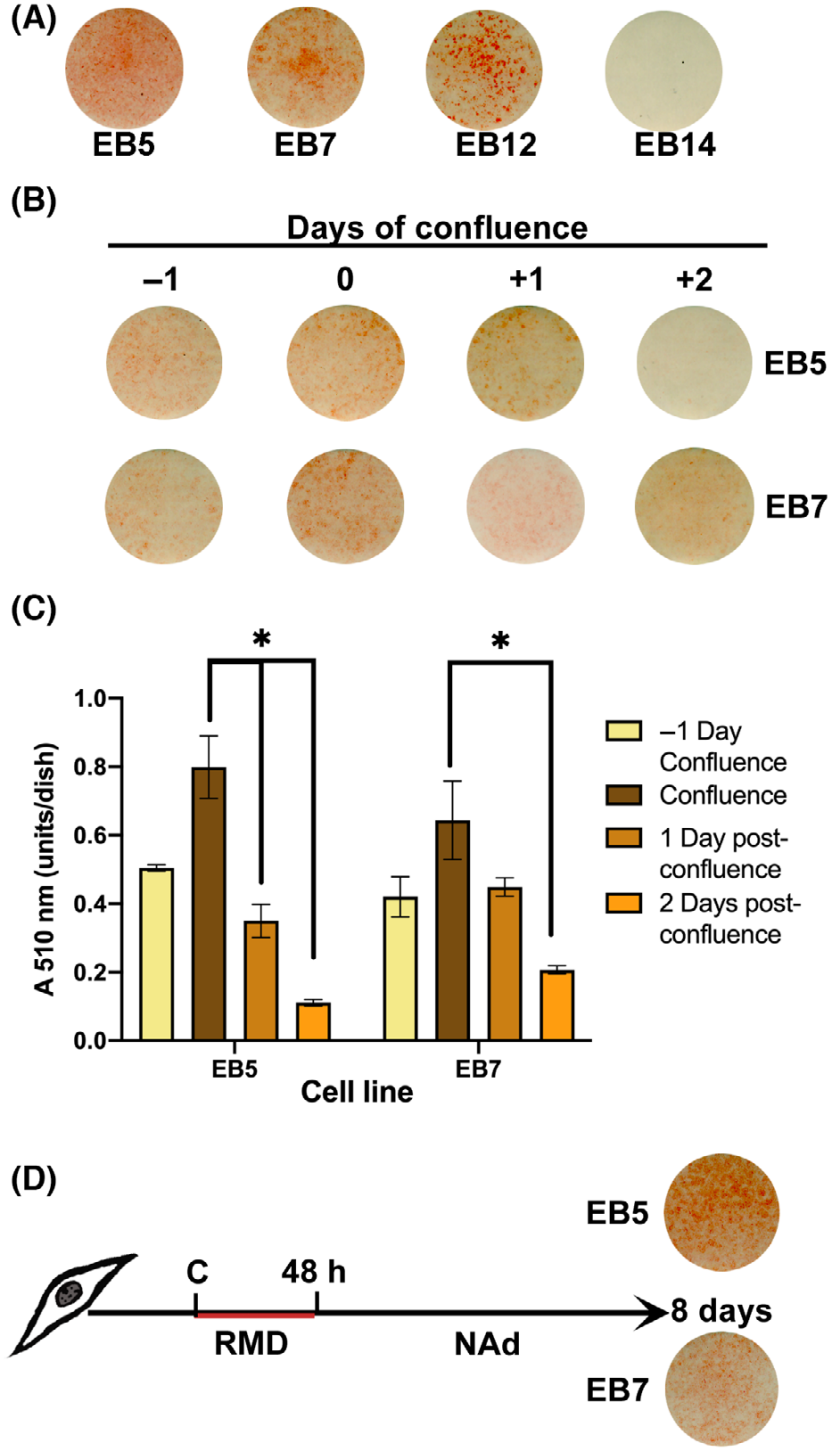
Murine embryonic clonal cell lines. Clones were cultured and induced to differentiation with RMD for 48 h and stained with Oil‐Red‐O at terminal differentiation to show lipid accumulation. (A) Four clones shown with different levels of lipid accumulation for comparative purposes, among them the EB5 and EB7 selected clones for their highest level of adipose differentiation. (B) The EB5 and EB7 cell cultures were stimulated at different days of confluence with RMD for 48 h to determine the optimal time of confluence for adipose induction. (C) Quantitation of Oil Red O staining of cultures shown in B. (D) The provided image is a schematic of the standardized cell culture protocol using RMD to induce adipogenesis, which was applied across all assays. NAd, nonadipogenic medium; RMD, 1 μm Rosiglitazone, 0.5 mm 3‐Isobutyl‐1‐methylxanthine, and 1 μm dexamethasone. The results are given as the mean SEM of two triplicate experiments (*n* = 6). ANOVA was used to establish statistical differences, indicated with an asterisk with a *P* value lower than 0.05.

After 48 h in RMD medium, we switched the cultures to a nonadipogenic medium. We proceeded to isolate 20 colonies exhibiting various degrees of adipose differentiation using cloning cylinders, following established protocols [36, 37]. These isolated colonies were then subcultured into 35‐mm tissue culture dishes. Prior to reaching confluence, cells were transferred to a 100‐mm tissue culture dish containing growth medium. Out of these, only two clones—designated as EB5 and EB7—were selected based on their elevated levels of adipose differentiation. This was determined by Oil Red O staining for intracellular lipid accumulation, as depicted in Fig. 1A. This figure also includes some of the other clones that demonstrated lower levels of adipose differentiation for comparative purposes.

In alignment with our previous work on F442A cells, which demonstrated that less than 48 h of induction with a differentiation cocktail is sufficient for cellular commitment to differentiation [51, 52], we adopted a similar approach for the three clones under study. The F442A cells were cultured in nonadipogenic medium (NAd), consisting of DMEM supplemented with 2.5% adult cat serum, 2.5% adult bovine serum, 5 μg·mL^−1^ insulin, and 1 μm d‐biotin; we previously found that cat serum is nonadipogenic for the F442A cells [53]. Two days after reaching confluence, the medium was switched to an adipogenic medium for 48 h. This adipogenic medium was DMEM supplemented with 4% adult cat serum, 1% adult bovine serum, 5 μg·mL^−1^ insulin, 5 μg·mL^−1^ transferrin, 1 μm d‐biotin, 2 nm triiodothyronine, and RMD.

For the EB5 and EB7 clones, they were initially grown in NAd medium, which included DMEM supplemented with 5% bovine calf serum that we found is nonadipogenic for these clones. Upon reaching confluence, we replaced the medium with adipogenic medium (comprising 5% bovine calf serum, 5 μg·mL^−1^ insulin, 5 μg·mL^−1^ transferrin, 1 μm d_−_biotin, and 2 nm triiodothyronine) containing RMD for a 48‐h period. Subsequently, the cultures were reverted back to NAd medium to undergo terminal differentiation, unless otherwise specified. All cultures were maintained at 37 °C in a 10% CO_2_ atmosphere. At the end of the experiment, cells were fixed overnight in 4% formalin and stained with Oil Red O to assess lipid accumulation [54].

To investigate the involvement of GSK3β in the differentiation process of the F442A, EB5, and EB7 clones, we conducted parallel experiments. The cells were cultured as previously described and were subjected to treatment with or without the GSK3β selective inhibitor, 100 μm SB415286, for 24 h during the adipogenic induction phase. The vehicle control cultures contained 1% DMSO.

For cold response assays, F442A, EB5, and EB7 cells were cultured until mature adipocytes formed, which occurred 8 days postdifferentiation induction. Following this, the culture medium was replaced, and after a 2‐h incubation, the cells were exposed to a 30 °C and 10% CO_2_ environment for 4 h. The cultures were then washed twice with PBS at 30 °C before being frozen.

### Mouse tissues

Male newborn C57BL/6 mice were sourced from the unit of production of experimental animals (UPEAL) at CINVESTAV‐IPN. Following cervical dislocation for euthanasia, in compliance with UPEAL‐CINVESTAV‐IPN institutional guidelines, tissues were promptly dissected and snap‐frozen in liquid nitrogen. Entire tissue depots were collected for analysis.

### Analysis of gene expression

We extracted total RNA with TRIzol® reagent, as described by the manufacturer. We transcribed 1 μg of total RNA into cDNA using oligodT primer, and SuperScript II™ reverse transcriptase reaction mix (Invitrogen, Carlsbad, CA, USA) according to the manufacturer's instructions. Relative quantitative real‐time PCR (RT‐PCR) was carried out with FastStart Universal SYBR Green I Master (Rox) kit (Roche Applied Science, Manheim, Germany). We monitored the reaction amplification on a CFX96™ Real‐Time PCR Detection System BIORAD (Bio‐Rad, Hercules, CA, USA). The specific primers for each gene are listed in Table 1. We determined the expression values using the $2^{-\Delta C_{T}}$ equation, and we normalized each gene expression to that of the ribosomal phosphoprotein large P0 (*Rplp0*) gene that was amplified from the same sample. In each experiment we compared each gene from the three clones, and we show gene expression as relative changes. The amplification products were separated in 1.5% (w/v) agarose gel. We verified the identity of all amplification products by sequencing, using an ABI PRISMTM 310 DNA Sequencer (PerkinElmer, Boston, MA, USA) and the Big Dye Terminator kit v3.1 (PerkinElmer). We compared the sequencing data with the reference GeneBank data [55] using the blastn 2.2.30 suite [56]. We used the names and acronyms of proteins and genes accordingly to the NCBI GenBank and GenPept databases (Table S1).

**Table 1:** Primers used for this study

Gene	Accession number	Sequence	*T*_a_ (°C)	Product size (bp)	Source
*Acacb*	NM_133904	CACCATTTTCAGCAAGCCCACTAT	61.4	157	This work
		GGCCGCACAGCTCATCTATCAG			
*Adipoq*	NM_009605	CAATGGCACACCAGGCCGTGAT	69.5	212	[95]
		CCAGCCCACACTGAACGCTGA			
*Atgl*	NM_025802	CAAGGGGTGCGCTATGTGGATGG	58	189	[57]
		GAGGCGGTAGAGATTGCGAAGGTT			
*Cd36*	NM_001159558	TGGCCTTACTTGGGATTGG	58	111	[57]
		CCAGTGTATATGTAGGCTCATCCA			
*Cebpa*	NM_007678	GAGTCGGCCGACTTCTACG	62	178	[64]
		GTCTCGTGCTCGCAGATGC			
*Cebpb*	NM_009883	CCGCGCACCACGACTTCCCCT	61	451	[64]
		CGCTCGCGCCGCATCTTGTA			
*Cidea*	NM_007702	TCATCAGGCCCCTGACATTC	57.4	186	This work
		CCAGGCCAGTTGTGATGACT			
*Cpt1b*	NM_009948	TTCCAAACGTCACTGCCTAAG	60	213	This work
		TTCCCACCAGTCACTCACATAG			
*Fabp4*	NM_024406	AAGAGAAAACGAGATGGTGACAA	62	65	[64]
		CTTGTGGAAGTCACGCCTTT			
*Fasn*	NM_007988	TTGGGGGCGTGAGATGTGTTG	60.9	106	This work
		CCTCGGGTGTGGTGGGTTTG			
*Gpd1*	NM_010271	CTGAGATCATCAACACTCAGC	60	111	[57]
		TGTCAGCGCCTGTTGCAGC			
*Lep*	NM_008493	GAGCGGGATCAGGTTTTGTGGT	64.5	161	[95]
		TGTCACTCTTTCCCGGTCTCTTCA			
*Lhx8*	NM_010713	GGCCCGCCATAAGAAACACG	64.4	203	This work
		TGGGGTAACAAGGGCTGGAGTC			
*Lipe*	NM_010719	TGCCCAGCGCCTGCTGACCA	*62*	*260*	[57]
		CGCCAGGCCAAGCAGGAGTCAAAC			
*Lpl*	NM_008509	ATGCAGAAGCCCCCAGTCGC	62	149	This work
		CCAGCTGGTCCACGTCTCCGA			
*Mlxipl*	NM_021455	AACGGAGGAAGAGCCCAGTGT	63.3	172	This work
		CGGAGCCGCTTTTTGTAGTAGA			
*Myf5*	NM_008656	AGGAGCTGCTGAGGGAACA	63	205	This work
		CTGGACACGGAGCTTTTATCTG			
*Ppargc1a*	NM_008904	TCCTCCCACAACTCCTCCTCATAA	63.4	171	This work
		GATTGCTCGGGCCCTTTCTTG			
*Pparg2*	NM_011146	TCGCTGATGCACTGCCTATG	60.0	60	[96]
		GAGAGGTCCACAGAGCTGATT			
*Prdm16*	NM_027504	CAGCACGGTGAAGCCATTC	60.0	149	This work
		GGGAGGAGGTAGTGCTGAACATCT			
*Rplp0*	NM_007475	AGGCCCTGCACTCTCGCTTTCTGG	60.0	347	[52]
		TGGTTCCTTTGGCGGGATTAGTCG			
*Scd1*	NM_009127	CGAGGGCTTCCACAACTACC	55.1	130	This work
		AACTCAGAAGCCCAAAGCTCA			
*Slc2a4*	NM_009204	ACCGGCTGGGCTGATGTGTCTGA	63	240	[57]
		TATGGTGGCGTAGGCTGGCTGTCC			
*Srebf1a*	NM_011480	TAGTCCGAAGCCGGGTGGGCGCCGGCGCCAT	60	106	[97]
		GATGTCGTTCAAAACCGCTGTGTGTCCAGTTC			
*Srebf1c*	NM_011480	ATCGGCGCGGAAGCTGTCGGGGTAGCGTC	63	116	[97]
		CTGTCTTGGTTGTTGATGAGCTGGAGCAT			
*Tcf21*	NM_011545	ACCTGACGTGGCCCTTTATGGT	60.3	230	This work
		GTGTTCTTGCGGGGTGGGATAG			
*Tmem26*	NM_177794	GTCATCCCACAGAGCCACCAAT	65.0	216	This work
		GCCGTCCCCACAAACATCAG			
*Ucp1*	NM_009463	GGATTGGCCTCTACGACTCA	60.4	380	This work
		TGACAGTAAATGGCAGGGGA			
*Zic1*	NM_009573	CTGGCTGCGGCAAGGTTTTC	63.2	208	This work
		GAGCTGGGGTGCGTGTAGGACT			

The gene names are displayed in italics.

The acronyms denoting the murine proteins and genes are according to the Mouse Genome Informatic (MGI).

### Lipolysis measurement

EB5 and EB7 preadipocytes were seeded at a density of 1 × 10^3^ cells·cm^−2^, and differentiation was initiated using RMD, as described above. After terminal differentiation, we switched to phenol red‐free DMEM supplemented with 5% calf serum, insulin, biotin, transferrin, and triiodothyronine. As lipolytic stimuli, we used 5 μm l‐Norepinephrine hydrochloride (Sigma‐Aldrich) for 7 h. We collected the media and used the Free Glycerol Determination Kit (Sigma‐Aldrich) to determine the free glycerol, following the manufacturer's instructions. We measured absorbance at 540 nm with a Biotech® Synergy™ HT reader (Agilent, Santa Clara, CA, USA). The data are expressed as mmoles of glycerol per mg of protein [57].

### Immunoblotting

We lysed the cultures in a buffer containing 50 mm Tris–HCl pH 7.5, 150 mm NaCl, 0.5% NP‐40, and 0.1% SDS, plus Complete® protease inhibitor (Roche Applied Science). We separated 70 μg of protein from the extracts using 12.5% SDS/PAGE and immunoblotted them with rabbit polyclonal anti‐UCP1 (AB23841; Abcam Limited, Cambridge, UK), as reported by Kiefer *et al*. [58] overnight at 4 °C and POX polyclonal goat anti‐Rabbit (111035003; Jackson ImmunoResearch Inc, WestGrove, PA, USA) for 1 h at room temperature. The proteins were evidenced by chemiluminescent HRP substrate Immobilon™ Western (Millipore, Billerica, MA, USA) and detected with the Odyssey® Fc Imaging System (LI‐COR Biosciences, Lincoln, NE, USA) image studio v. 5.2.

### Data presentation and statistical analysis

Data are presented as mean ± standard error, derived from six or more cultures across two to three separate experimental runs. Statistical comparisons were made using a two‐tailed Student's *t*‐test for two groups and analysis of variance (ANOVA) for three or more groups, utilizing graphpad prism v. 9.0.1 (GraphPad Software, Boston, MA, USA) for macOS. A *P* value of ≤ 0.05 was considered statistically significant. Qualitative data corresponds to one representative experiment done in triplicate.

## Results

### The EB5 and EB7 cells express gene markers for brown and brite fat cell lineages, respectively

Most of the studies promote adipose differentiation by adding the adipogenic cocktail at 2 days after confluence. The two clones EB5 and EB7, isolated as described in Methods, undergo adipose differentiation by adding RMD in the culture medium. The optimal time to add RMD for 48 h was the confluence day, since induction 1 day before, or 1 or 2 days after confluence, resulted in a lower differentiation than the day of confluence. Quantitation of Oil Red staining of intracellular lipids [54] correlated with the stained culture dishes (Fig. 1A–C). After incubation with RMD, the cultures were kept with NAd for an additional 6 days until terminal differentiation (Fig. 1D).

**Fig. 2:**
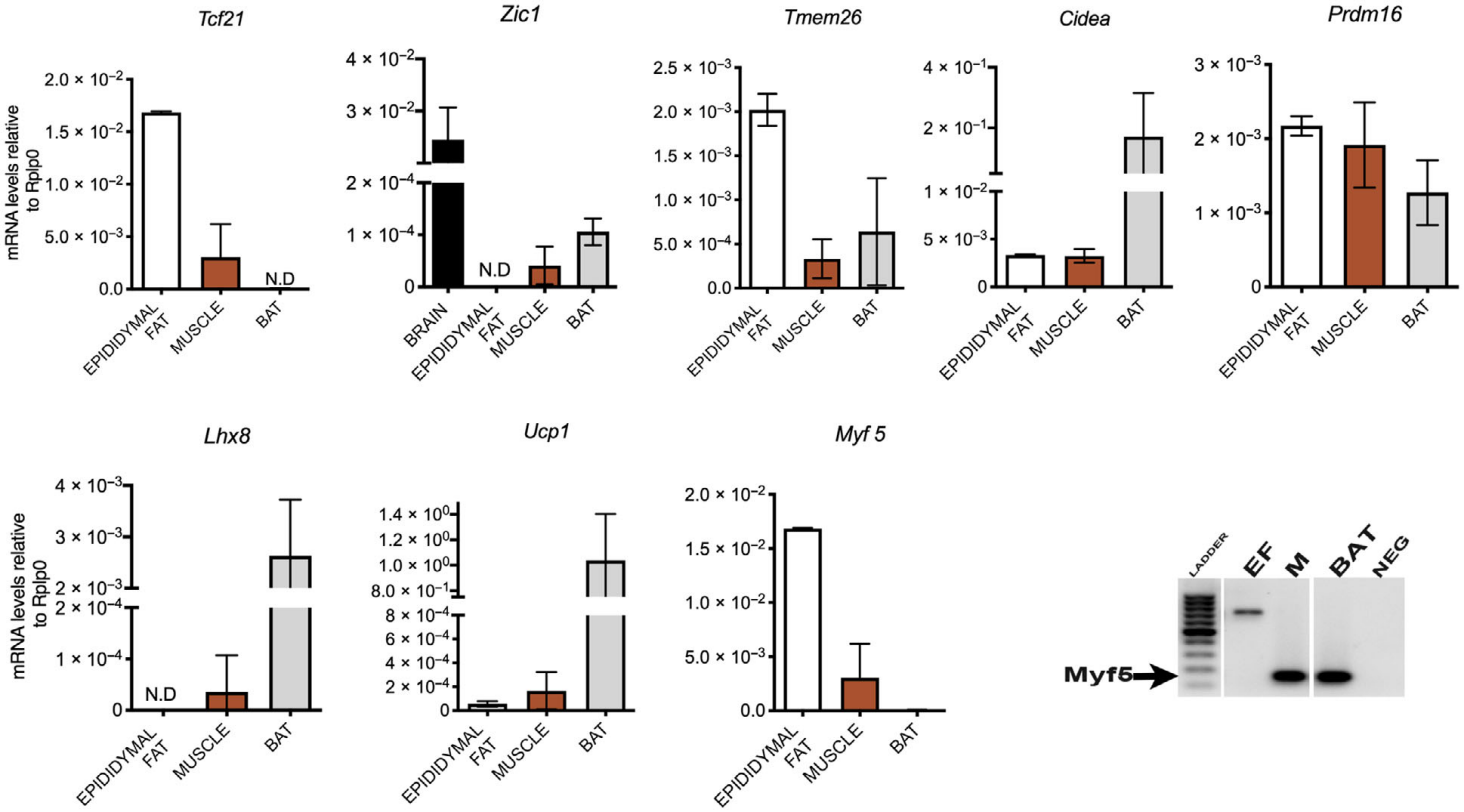
Expression of the genes widely accepted as typical markers for brown, brite, and white adipose tissues. RNA was extracted from mouse tissue samples, brain, muscle (M), epididymal (EF), and brown fat (BF), and we carried out RT‐PCR to determine gene expression. The *Myf 5* amplicon band is shown in the gel electrophoresis panel ThermoFisher ultra‐low‐range DNA ladder is shown in lane 1. The results are given as the mean SEM of two experiments.

To identify the adipose phenotype of the EB5 and EB7 clones we determined, by RT‐PCR, the expression of the genes widely accepted as typical for each adipose tissue, as previously described [38, 39]. We determined by RT‐PCR the expression of the lineage genetic markers *Tcf21* (white), *Zic1* (brown), *Myf5* (brown fat progenitors) [2], *Lhx8* (brown), *Tmem26* (brown and brite), *Prdm16* (brown and brite), and the expression of brown and brite fat functional genes, Cell death activator *Cidea*, and *Ucp1* [38, 39]. We extracted the RNA from iBAT; eWAT for brite and white fat genes. Yet, eWAT has precursors of brite adipocytes and undergo britening by cold exposure or when adipocytes in culture are treated with rosiglitazone [38, 49, 59]. Figure 2 shows that each gene was expressed according to the tissue reported data. *Myf5* seemed to be expressed in eWAT and muscle; however, sequencing of each amplicon showed that the band in both muscle (M) and brown fat (BF) was indeed a *bona fide Myf5* band, but not that in eWAT (Fig. 2, gel electrophoresis panel of amplicon). For *Zic1* we used brain tissue as a control to validate gene expression, consistent with published data indicating high levels of *Zic1* in brain.

The expression of some of the brown and brite genes in eWAT might be due to some britening of eWAT during tissue manipulation. Then we determined by RT‐PCR the expression of genes in each clone regarded as typical genes expressed in muscle and epidydimal and brown adipose tissues (Fig. 2) [38, 39].

**Fig. 3:**
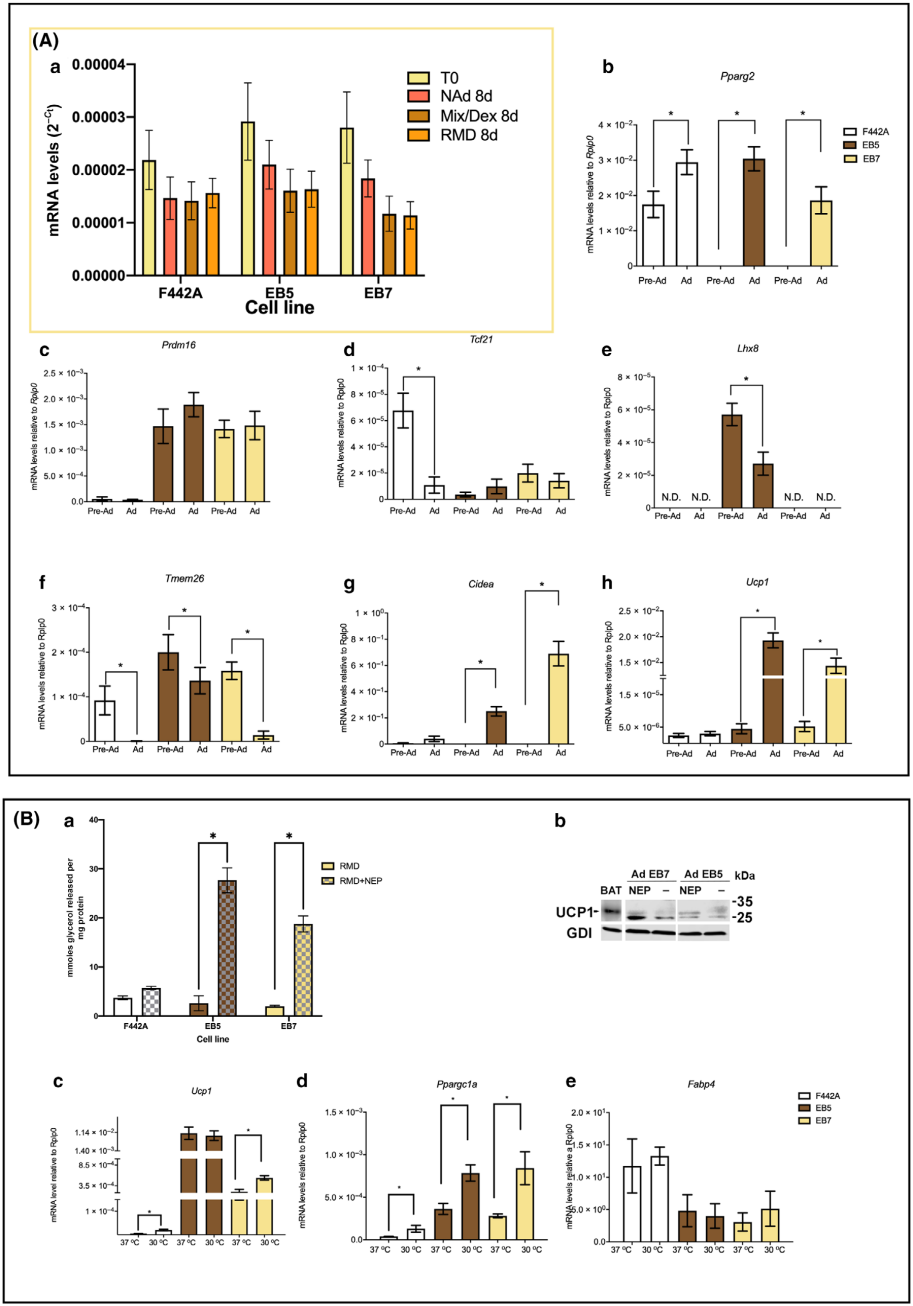
(A) Adipose marker gene expression from EB5, EB7, and F442A cells. EB5 and EB7 cultures were treated at confluence, and F442A at 2 days postconfluence, for 48 h with NAd, Mix/Dex, or RMD, and gene expression was measured using RT‐PCR. (a) Expression of *Rplp0*; (b) Expression of *Pparg2*; (c–h) Expression of the lineage specific markers *Prdm16*, *Tcf21*, *Lhx8*, *Tmem26*, *Cidea*, and *Ucp1*. (B) Physiological response to β_3_‐adrenergics and gene expression after cold exposure. (a) Lipolysis assay. Adipocyte cultures were incubated for 7 h with 5 μm l‐norepinephrine hydrochloride. Lipolysis was measured as glycerol release. (b) UCP1 WB. Seventy micrograms of proteins obtained from cultures stimulated with 5 μm l‐norepinephrine hydrochloride was used to immunoblot UCP1. (c–e) Cold response. Adipocyte cultures induced with RMD were incubated at 30 °C for 4 h, and the expression of *Ucp1*, *Ppargc1a*, and *Fabp4* genes was measured using RT‐PCR. The results are given as the mean SEM of two triplicate experiments (*n* = 6). Asterisks indicate statistical differences with a *P* value ≤0.05, evaluated by ANOVA, except for *Lhx8* where *T* student was used.

Peroxisome proliferator activated receptor gamma 2 (*Pparg2*) expression was used as the adipogenic gene marker. We chose *Rplp0*, the ribosomal phosphoprotein large P0 protein, as the reference gene since it did not show any statistically significant difference in the three clones under the used culture conditions (Fig. 3Aa).

The three cell lines cultured in NAd medium and induced with RMD highly expressed the adipocytes marker *Pparg2*, whereas those preadipocytes cultured with NAd did not express *Pparg2* (Fig. 3Ab). *Prdm16*, a brown and brite marker gene, was equally expressed in EB5 and EB7 preadipocytes and adipocytes, but not expressed in F442A (Fig. 3Ac). *Tcf21* expression was low in EB5 and EB7 preadipocytes and adipocytes, but high in F442A preadipocytes, but not in adipocytes (Fig. 3Ad). The EB5 preadipocytes expressed the widely accepted brown marker *Lhx8* that was downregulated in the adipocytes, but its expression was undetectable in the EB7 and F442A preadipocytes and adipocytes (Fig. 3Ae). *Tmem26* was highly expressed in EB5 and EB7 preadipocytes, EB5 adipocytes expressed *Tmem26* greater than EB7 adipocytes, and F442A preadipocytes, but not adipocytes expressed *Tmem26* (Fig. 3Af). EB5 and EB7 adipocytes, but not preadipocytes, and neither F442A preadipocytes nor adipocytes, expressed *Cidea* and *Ucp1*, the thermogenic genes (Fig. 3Ag,h). In all clones, *Myf5* and *Zic1* expression was undetectable. Together, these results strongly suggested that EB5 are brown preadipocytes, since they express *Lhx8*, a brown selective gene. The EB7 are brite preadipocytes, since they did not express *Lhx8*, but they did express *Tmem26*, a brown and brite selective gene. Both EB5 and EB7 adipocytes expressed *Ucp1* and *Cidea*. The F442A preadipocytes are of the white adipose lineage and they expressed *Tcf1*, but did not express *Cidea* and *Ucp1*. Since *Myf5* and *Zic1* expression was undetectable, we did not explore the mechanisms of the early progenitor development, since by the cloning methodology, most likely, the isolated clones have already passed the early progenitor stages. For example, EB5 cells represent initial‐stage brown preadipocytes, having successfully transitioned beyond the progenitor phase, characterized by the expression of *Myf5* and *Zic1*, but may be positioned within a subsequent phase of development, distinguished by the expression of the brown preadipocyte marker *Lhx8*. As for the cellular progenitors of the ‘brite’ lineage, it has been observed that these cells express *Prdm16* [60], a trait similarly exhibited by the EB7 clone (akin to EB5). This pattern could indicate that EB7 cells constitute early‐stage brite preadipocytes.

#### Physiological response to norepinephrine of the EB5 and EB7 adipocytes

We treated the EB5 and EB7 adipocytes with 5 μm norepinephrine and determined lipolysis by a glycerol release assay and UCP1 content by immunoblotting, as a response to the β_3_‐adrenergic stimulation. Norepinephrine stimulated lipolysis by at least 18‐fold in the EB5 and EB7 adipocytes relative to untreated cells; but it did not significantly stimulate the F442A white adipocytes (Fig. 3Ba). Immunoblotting of UCP1 by a specific antibody revealed that norepinephrine concomitantly increased UCP1 protein content in EB5 and EB7 adipocytes (Fig. 3Bb). Our results align with previous observations that showed norepinephrine increased oxygen consumption, expression of *Ucp1* mRNA, and lipolysis, as described above (see Introduction). Our results point out that EB5 and EB7 adipocytes are physiologically competent.

### The response to cold supports that EB5 are brown and EB7 are brite adipocytes

Interscapular brown fat in mice exposed to cold (4 °C) temperatures for 2 h increased the expression of *Ppargc1a* but not of *Ucp1* [61]. The 3T3‐L1 and F442A white adipocytes and a cultured brite adipocyte clones exposed to 27–33 °C for 4 h increased *Ucp1* and Peroxisome proliferative activated receptor gamma coactivator 1 alpha (*Ppargc1a*) expression, responding to cold stimuli, while a brown fat clonal line did not; cold also did not increase cytosolic Fatty acid binding protein 4 (*Fabp4*) gene expression [8, 40]. Based on our genetic marker data, we have inferred that the EB5 clone is derived from a brown fat lineage, whereas the EB7 clone is associated with the brite fat lineage. To confirm the identification suggested by the genetic markers, we conducted experiments to assess the expression of functional genes, *Ucp1*, *Ppargc1a*, and *Fabp4*, in response to cold exposure, as previously described by Ye *et al*. [40]. We incubated the adipocyte cultures at 30 °C for 4 h. Cold incubation increased *Ucp1* expression in F442A and EB7 adipocytes, and increased *Ppargc1a* expression in the three types of adipocytes. While cold did not increase *Ucp1* expression in EB5 adipocytes, it is worth noting that *Ucp1* expression was already elevated in these cells prior to cold exposure. Under unstimulated conditions, brown adipocytes have relatively higher levels of *Ucp1* than beige adipocytes. Cold had no effect on *Fabp4* expression in any of the three adipocytes studied (Fig. 3Bc–e). These results, integrated with those on the lineage gene expression described above, support the conclusion that the EB5 clone is indeed of the brown lineage and EB7 of the brite lineage.

### Mix/Dex and St/Dex induce commitment to differentiation, and rosiglitazone stimulates the brown and brite adipose phenotype expression

The study of induction or commitment to differentiation into adipocytes is paramount to understanding the early mechanisms of adipogenesis. Small drug‐like molecules added to cells cultured in NAd medium can facilitate the study of the early expression of genes and molecular pathways leading to commitment. For example, they offer the advantage of altering signaling pathways that are otherwise regulated by endogenous serum adipogenic proteins. Differentiation of the white 3T3‐L1 and F442A cells depends on adipogenic serum supplemented to the culture medium [53]. Mix/Dex are a common cocktail that enhances adipogenesis in cells cultured in medium supplemented with adipogenic serum [62]. Mix inhibits phosphodiesterases, and blocks the inhibitory regulatory protein G_i_ and stimulates adenyl cyclase activity [63]. Furthermore, Staurosporine (St) rapidly induced commitment to differentiation of F442A cells during only 4 h incubation in nonadipogenic or serum‐free medium; Dex did not induce it but enhanced adipose conversion [52, 64]. St, a serine/threonine kinase inhibitor, activates GSK3β by promoting its dephosphorylation in Ser^21/9^ [65]. Mix/Dex combined with rosiglitazone (Ros), an agonist of PPARγ, have been used to differentiate brown and brite preadipocytes. Ros stimulates “browning” in white subcutaneous adipose tissue and in primary cultures of adipocytes from murine BAT [66, 67]. However, no studies have been done with Mix/Dex or St/Dex to induce differentiation of brown or brite cells cultured in NAd or serum‐free medium.

**Fig. 4:**
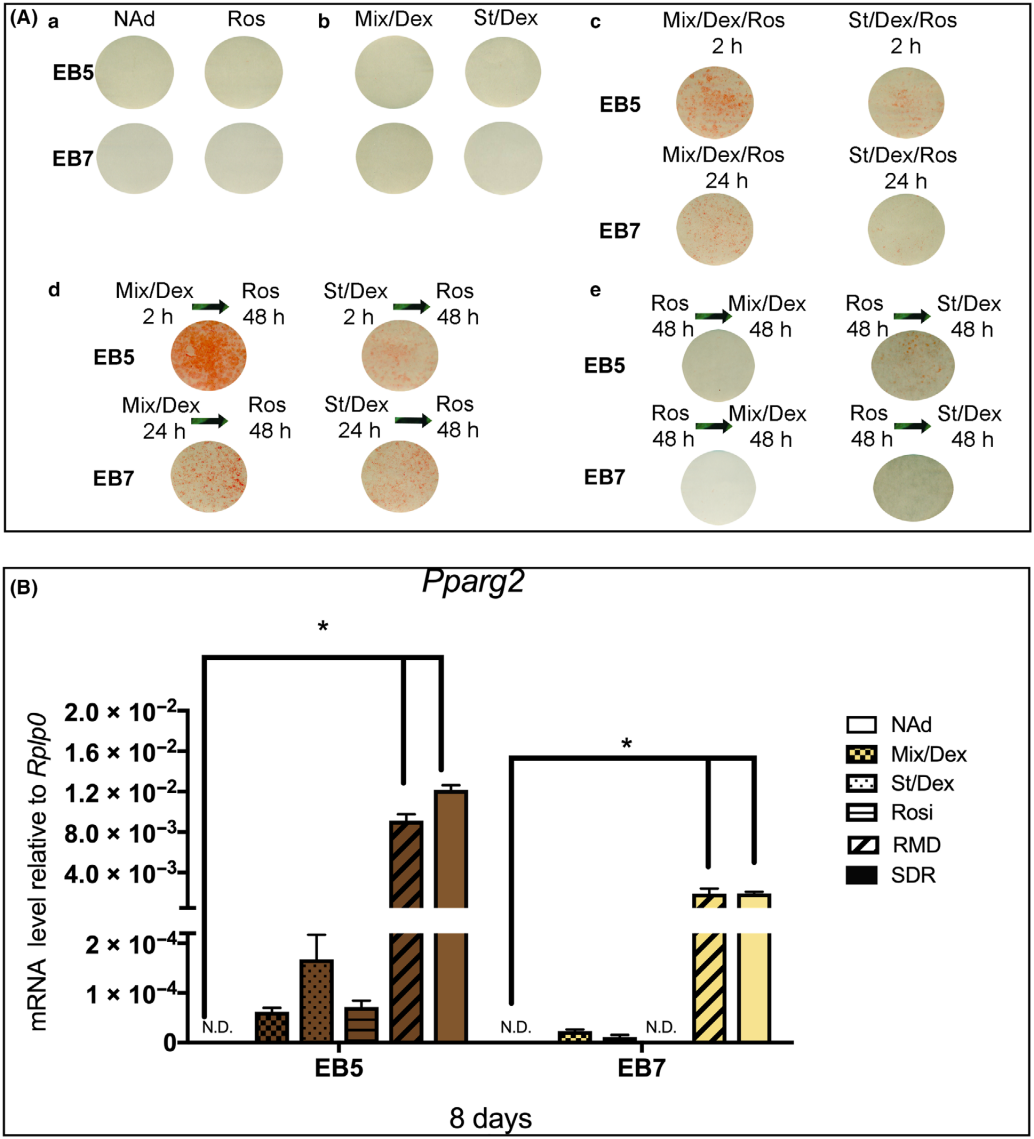
(A) Effect of Mix/Dex, St/Dex, and Ros on commitment to differentiation. (a) Representative photographs of adipose conversion from confluent cultures of EB5 and EB7 cells treated for 48 h with NAd or Ros. Cultures were followed up to 192 h and stained with Oil Red O; (b) Similar cultures as in a, but treated with Mix/Dex or St/Dex; (c) Confluent cultures induced by 2 h for EB5 and 24 h for EB7 with RMD or SDR; (d) Confluent cultures induced by 2 h for EB5 and 24 h for EB7 with Mix/Dex or St/Dex and then stimulated for 48 h with Ros; (e) Cultures treated by 48 h with Ros and stimulated with Mix/Dex or St/Dex for 24 h. (B) Cultures, treated for 48 h with each stimulus, were harvested at 192 h. Gene expression was assessed by RT‐PCR. The data are presented as the mean ± SEM of two triplicate experiments (*n* = 6). Asterisks indicate statistical differences between the indicated data groups with a *P* value ≤0.05, evaluated by ANOVA.

We thought it of interest to induce commitment of brown and brite adipocyte differentiation with these small molecules. RMD or St/Dex/Ros (SDR) induced rapidly the differentiation of the EB5 and EB7 cells cultured in NAd medium; EB5 were induced by 2 h and B7 by 24 h (Fig. 4Aa–c). The EB5 and EB7 cells treated with Mix/Dex, St/Dex, or Ros alone did not undergo morphological changes or accumulate intracellular lipids, and they remained with a fibroblastic morphology (not shown), as did cells cultured in NAd (Fig. 4Aa,b). However, EB5 and EB7 cells first treated with Mix/Dex or St/Dex, as described above, and then followed with only Ros for up to 48 h underwent adipose differentiation (Fig. 4Ad), while cells treated with Ros for 48 h and then with Mix/Dex or St/Dex did not differentiate (Fig. 4Ae).

We examined the expression changes of *Pparg2* in cells treated with RMD or SDR. *Pparg2* highly increased its expression in differentiated adipocytes of both EB5 and EB7 cells (Fig. 4B). Parallel cultures treated with Mix/Dex or St/Dex without Ros or only with Ros did not express *Pparg2*, similar to cells cultured in NAd medium (Fig. 4B). Altogether, these results showed that Mix/Dex and St/Dex induced commitment or primed the EB5 and EB7 cells to undergo adipogenesis, remaining with fibroblastic morphology, but they needed Ros to undergo phenotypic expression and terminal differentiation into adipocytes. Ros by itself did not induce differentiation. Since this adipose model can separate Mix/Dex or St/Dex from Ros action, it provides valuable information needed to study early genes expressed during preadipocyte commitment and later genes expressed in the brown or brite adipocyte.

### The adipogenic transcriptional cascade in brown, brite, and white adipocytes

Differentiation of white preadipocytes involves a complex activity of transcription factors; including CCAAT/enhancer binding protein (C/EBP) beta (CEB/Pβ), PPARγ2, CCAAT/enhancer binding protein alpha (CEB/Pα), Sterol regulatory element‐binding protein 1 isoform c (SREBP1c) [60, 68, 69], and some members of the Kruppel family of transcription factors [70, 71, 72]. We previously reported a temporal relationship in the expression of the genes encoding the transcription factors during F442A cells adipogenesis [64]. *Srebf1a* was expressed shortly after *Cebpb* and silencing of *Srebf1a* blocked adipose differentiation as well as the expression of *Pparg2*, *Cebpa*, and *Srebf1c*, indicating its crucial role during white adipogenesis [64]. The brown and brite adipogenic gene expression is not fully studied. Therefore, we examined the gene expression in EB5 and EB7 cells during differentiation at various culture times.

**Fig. 5:**
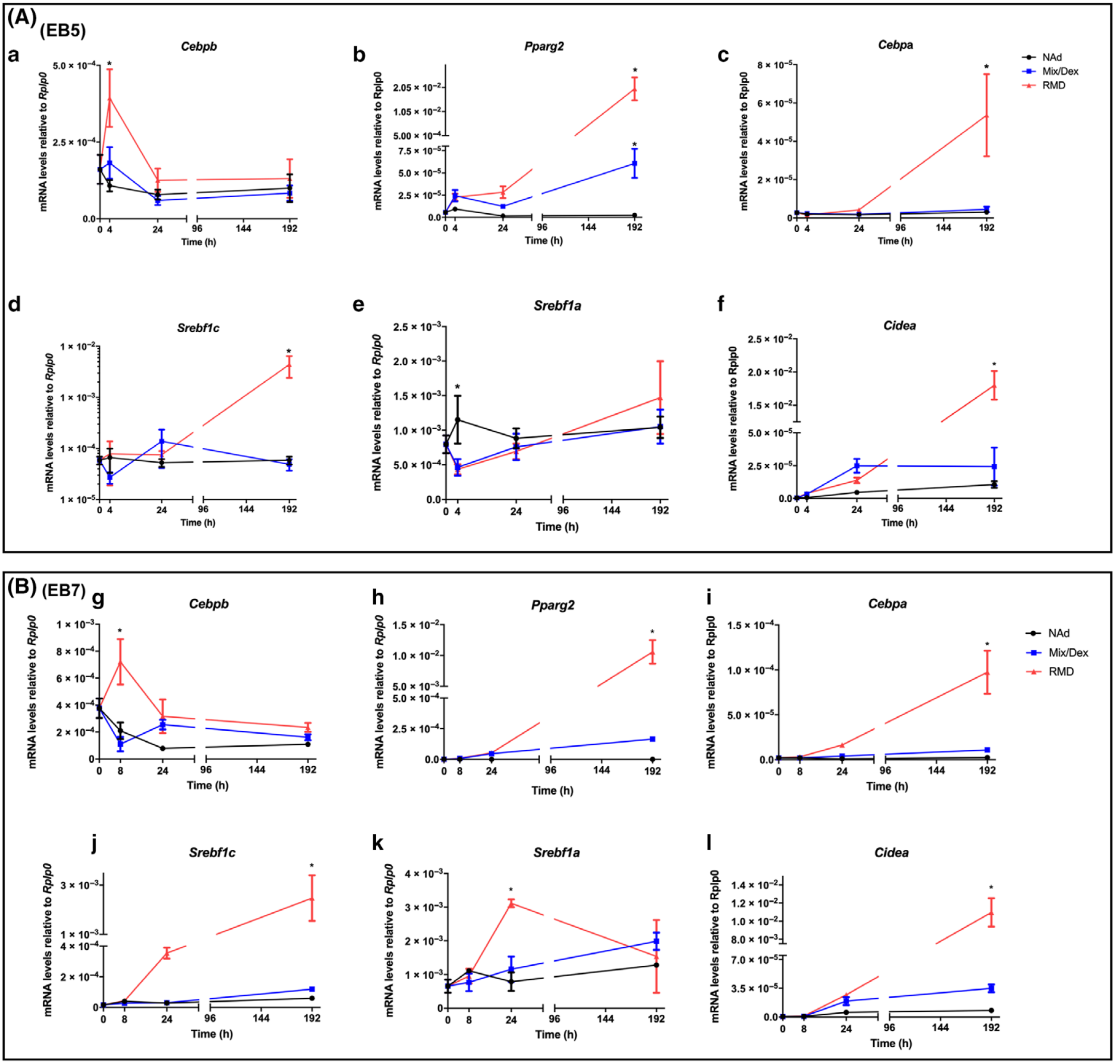
Expression of adipogenic genes along the culture time in EB5 brown and EB7 brite cell clones. (A) EB5 cells. (B) EB7 cells. Confluent cultures were treated for 48 h with NAd, Mix/Dex, or RMD, and gene expression was measured using RT‐PCR at various timepoints. The results are given as the mean ± SEM of two triplicate experiments (*n* = 6). Asterisks indicate statistical differences with a *P* value ≤< 0.05 between the data groups, evaluated by ANOVA.

The time‐course expression of *Cebpb*, *Pparg2*, *Cebpa*, and *Srebf1c* in EB5 (Fig. 5A) and EB7 (Fig. 5B) cells treated with RMD was similar to that seen in F442A cells cultured in adipogenic conditions [64]. *Cebpb* was expressed early and transiently, reaching its maximum at 4 and 8 h for EB5 and EB7, respectively (Fig. 5Aa,Bg). *Pparg2*, *Cebpa*, and *Srebf1c* expression began later than that of *Cebpb*, reaching its maximum at the mature adipocyte state in both cell lines (Fig. 5Ab–d,Bh–j). *Srebf1a* expression did not increase in EB5, but it significantly did in EB7 at 24 h before decreasing to the NAd low levels in the adipocytes (Fig. 5Ae,Bk); *Srebf1a* increased its expression early in F442A cells and remained high up to terminal differentiation [64].

CIDE‐A is a lipid droplet protein that is shuttled to the nucleus, where it acts transcriptionally to drive UCP1 expression through promoting PPARγ binding to a *Ucp1* enhancer element in human adipocyte britening [73]. CIDE‐A gene expression increased after the genes of the adipogenic transcriptional cascade and well during adipose terminal differentiation in EB5 and EB7 cells (Fig. 5Af,C). *Ucp1* was also expressed at the adipose stage (Fig. 3Ah). The results showed the expression of the genes encoding some of the key transcription factors during white adipocyte differentiation occurs in the EB5 and EB7 cells as well. The most significant difference was the expression of *Srebf1a*, which did not seem to have a crucial role in brown, but it likely does in brite fat adipogenesis, as in white fat cells. EB5 and EB7 cells treated with Mix/Dex without Ros did not express any of these genes (Fig. 5Ae,Bk), accordingly to our data described above.

### GSK3β regulates EB5 and EB7 adipogenesis

**Fig. 6:**
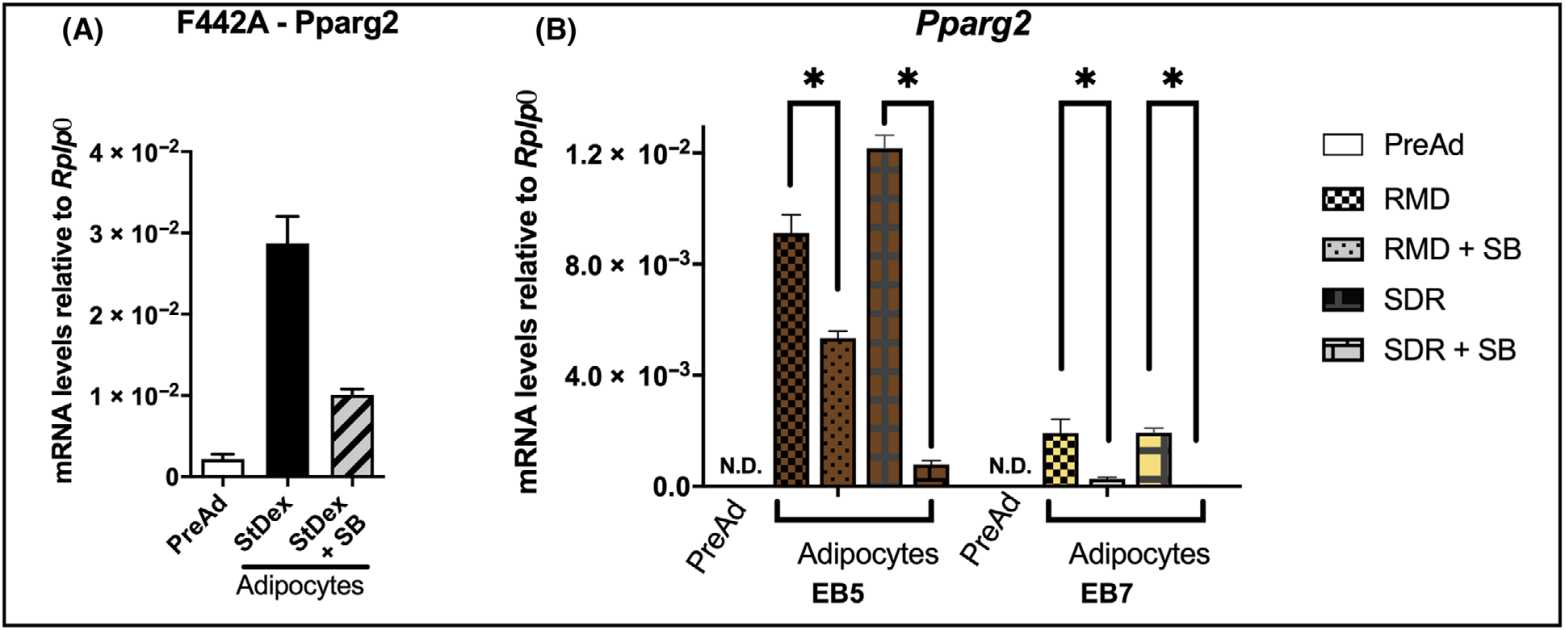
SB415286 inhibits adipogenesis. (A) Postconfluent F442A cultures treated for 8 h with NAd, St/Dex, or St/Dex with SB415286 100 μm. (B) Confluent cultures of EB5 or EB7 cells were treated for 48 h with NAd, SDR, or RMD with or without SB415286. *Pparg2* gene expression was measured using RT‐PCR. The results are given as the mean ± SEM of two triplicate experiments (*n* = 6). Asterisks denote a *P* value ≤ 0.05 between the two groups, evaluated by ANOVA.

GSK3β is a well‐described regulator of adipogenesis and the transcriptional cascade in white adipogenesis [64,74] and has been proposed to be a negative regulator of the adrenergic‐mediated thermogenic program in brown adipocytes [75]. The role of GSK3β in brite cells has not been reported. We interrogated whether activation of GSK3β is also a crucial early event in the brown and brite transcriptional pathways. We cultured the EB5 and EB7 cells as described above, and at confluence, we induced the cultures with RMD or SDR. We treated parallel cultures with the selective blocker of GSK3β activity SB415286 for 24 h, and we measured gene expression. We also treated the F442A preadipocytes as a comparative white fat cell model. Since *Pparg2* is stably expressed after 24 h, we determined its expression in terminally differentiated adipocytes. As we previously showed 64, SB415286 inhibited *Pparg2* expression in F442A adipocytes (Fig. 6A). *Pparg2* expression was high in both EB5 and EB7 cells induced with RMD or SDR, as expected (Fig. 6B), and SB415286 inhibited it in EB5 and EB7 cells more when we induced the cells with SDR (about 90%) than with RMD (about 50%) (Fig. 6B). These results show that GSK3β activity is required for EB5 and EB7 as it is for F442A adipose differentiation, implying that this enzyme has a regulatory role in brown and brite adipogenesis, leading to the transcriptional adipogenic cascade expression.

### Expression of lipid and carbohydrate metabolism genes in EB5 and EB7 adipocytes

#### Genes commonly expressed in the brown, brite, and white adipocytes: glucose and fatty acid uptake, lipogenesis, lipolysis, and intracytoplasmic fatty acid transport

**Fig. 7:**
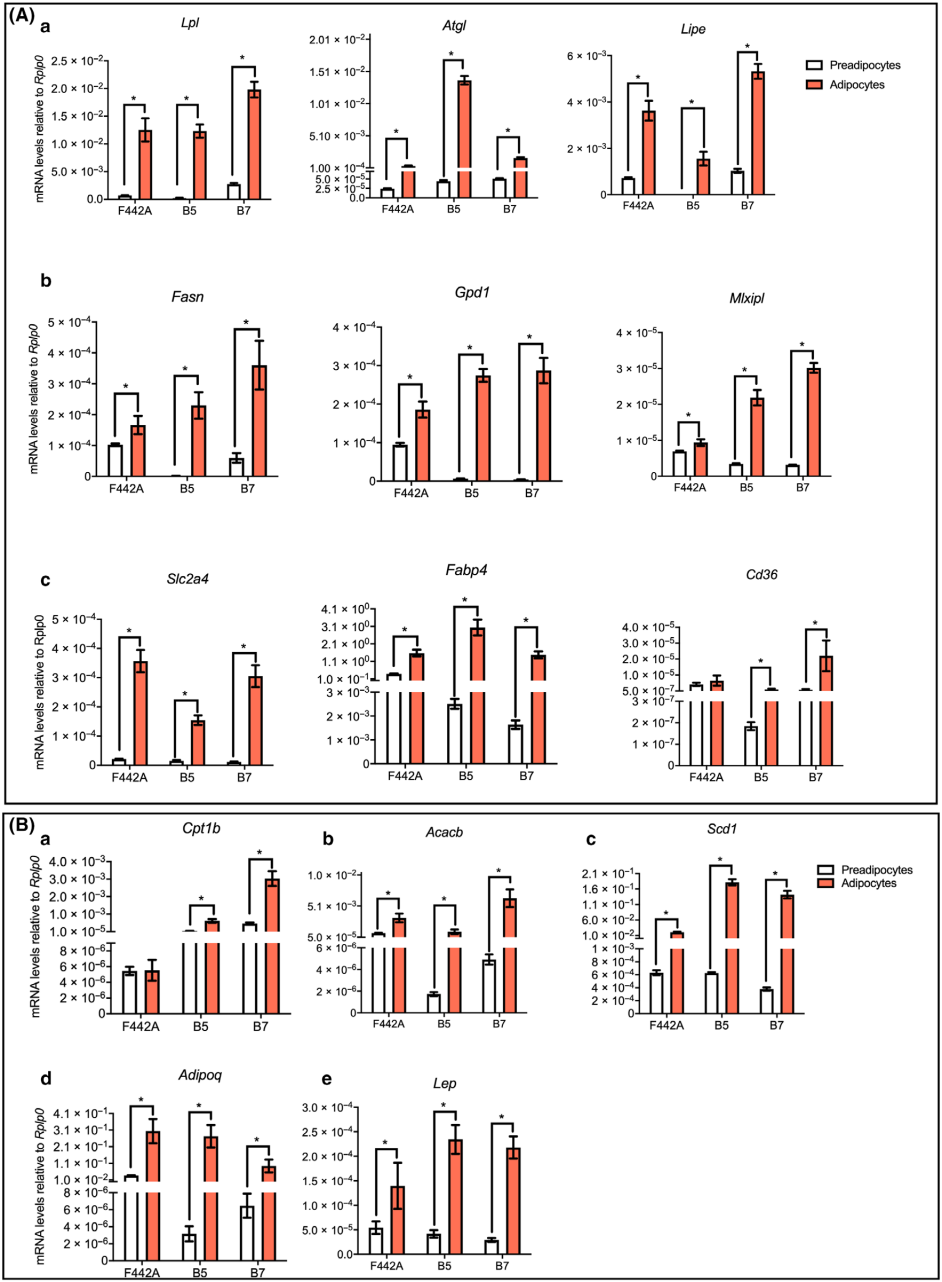
(A) Expression of genes related to beta‐oxidation and MUFA synthesis. Postconfluent cultures of F442A cells or confluent cultures of EB5 or EB7 cells were treated with NAd or RMD for 48 h, and gene expression was measured using RT‐PCR after 192 h. (B) Expression of lipid and glucose metabolism genes and adipokines. Cultures treated as in panel A. The results are given as the mean ± SEM of two triplicate experiments (*n* = 6). Asterisks indicate statistical differences with a *P* value ≤ 0.05 between the indicated data groups, evaluated by ANOVA.

*Lpl*, *Atgl*, *Lipe*, *Fasn*, *Gpd1*, and *Mlxipl* were all highly expressed in the adipocytes of the three clones when compared with the preadipocytes, as expected (Fig. 7Aa,b). *Slc2a4* and *Fabp4* increased their expression in all three adipocytes compared to the preadipocytes, but *Cd36* only in EB5 and in EB7 but not in F442A adipocytes (Fig. 7Ac).

#### Genes selectively expressed in brown and brite adipocytes

##### Beta‐oxidation

Relative to preadipocytes, *Cpt1b* expression increased about 1000‐fold in the adipocytes of EB5 and EB7, but not in F442A adipocytes (Fig. 7Ba).

*Acacb* increased its expression in the adipocytes in comparison with the preadipocytes in the three clones. While it only increased about 10‐fold in F442A adipocytes, it increased by more than 1000‐fold in EB5 and EB7 adipocytes (Fig. 7Bb), indicating it plays a crucial role in the brown and brite fat oxidation.

##### Mono‐unsaturated fatty acid synthesis

*Scd1* expression increased in EB5 and EB7 adipocytes, by about 1000‐fold compared with preadipocytes, and only 10‐fold in F442A adipocytes compared to preadipocytes (Fig. 7Bc), implying that brown and brite adipocytes would have a more active synthesis of MUFA than white adipocytes.

##### Adipokines

*Adipoq* (adiponectin) (Fig. 7Bd), and *Lep*, the gene encoding leptin (Fig. 7Be), increased their expression in EB5, EB7, and F442A adipocytes compared to their corresponding preadipocytes.

These results show that the EB5 and EB7 adipocytes have the metabolic pathways required for brown and brite fat cells to transport glucose and to provide fatty acids from different sources for mitochondrial beta‐oxidation and energy expenditure, as seen in BAT and BRT. Additionally, we can expect that they are also engaged actively in the synthesis and signaling of both adipokines.

## Discussion

Prior to our studies, it was not clear if brite progenitors arise during embryonic stages or after birth.

Brite adipocytes are activated after birth or during adulthood. As amply reviewed by Shao *et al*. [76], studies by different researchers in animal models have suggested, still controversially, that brite fat might arise via two possible mechanisms: (a) A white‐to‐brite fat and a brite‐to‐white transition, giving some support to the idea that brite adipocytes might remain dormant with a unilocular white adipocyte morphology. These results suggest that the differentiated adipocytes might have interchangeable morphologies under certain physiological or pharmacological conditions. (b) Specific precursors that undergo *de novo* differentiation into brite adipocytes after cold exposure, as supported by genetic tracing and lineage experiments.

Gupta and colleagues [76] recently proposed that brite adipocytes arise from cell progenitors through *de novo* brite adipogenesis during the first cold exposure. Subsequently, the cells interconvert between the dormant and the active brite phenotypes, a process called beige cell activation, in response to physiological or pharmacological stimulation. The interconversion between the dormant and the brite adipose phenotypes offer the benefit to rapidly respond to environmental changes [76]. Since we isolated the beige EB7 clone from 11–12 days mouse embryos, we demonstrated that cell progenitors for brite adipogenesis arise during early embryonic life. This is around the same time as the brown adipocytes and earlier than white fat arises. We suggest that the brite adipose progenitors are well established during embryonic life, before the first cold exposure to activate brite fat. We cannot conclude if theses progenitors undergo adipose differentiation in the embryo and then remain dormant as beige fat, or if they undergo adipogenesis after birth and the first cold stimulation.

As reported earlier, 4 h exposure to cold temperatures stimulated the expression of *Ucp1* and *Ppargc1a* directly at the cellular level in white and beige adipocyte cell lines, but not in brown adipocyte cell lines [40]. In contrast, 10 °C cold exposure for 20 h induced *Ucp1* and *Ppargc1a* expression in the intrascapular BAT in mice [40], whereas mice exposed to 4 °C for 2 h increased *Ppargc1a* expression but not *Ucp1* expression in the interscapular BAT [61]. The discrepancy between these published *in vitro* and *in vivo* studies remains unresolved, but may be related to the different times and temperatures used in these studies. In our experiments, cold incubation of the EB7 adipocytes stimulated *Ucp1* and *Ppargc1a* expression, consistent with a beige phenotype for EB7 cells. Cold exposure of EB5 adipocytes increased *Ppargc1a* but not *Ucp1* expression, which is consistent with the *in vivo* studies of BAT [61]. However, this is in contrast to the immortalized brown clonal adipocytes isolated from the stromal vascular fraction, where cold‐induced *Ppargc1a* was not observed [40]. This discrepancy might be explained by clonal cell differences and/or the inherent heterogeneity of adipocyte populations, such as differences in genomic imprinting and/or temporal regulation of gene expression. This is not surprising, given that single‐cell RNA sequencing in murine BAT cells showed distinct brown preadipocyte and adipocyte populations, indicating the presence of heterogenous adipocytes. For example, *Ucp1* expression greatly varies within individual adipocytes in morphologically uniform murine BAT [77]. Indeed, unstimulated EB5 cells expressed far more *Ucp1* than unstimulated EB7 cells and, as pointed out by Wang and Seale [78], under unstimulated conditions, brown adipocytes express relatively higher levels of UCP1 than beige adipocytes. Therefore, the data integration of the genetic markers expression and the *Ucp1*, *Ppargc1a*, and *Fabp4* expression in response to cold support the conclusion that EB5 is of the brown fat lineage, and the EB7 is of the brite lineage.

### Induction of adipogenesis

Cell differentiation is characterized by commitment, clonal expansion, and expression of the new phenotype (i.e., mature adipocytes). We attempted to study the molecular events leading to the induction of brown and brite adipose commitment using small molecules that act intracellularly, circumventing the effect of serum adipogenic substances or other proteins on the cell surface. In the NAd medium, we promoted differentiation with either of two adipogenic cocktails: Mix/Dex/Ros and St/Dex/Ros. Because Ros in combination with Mix/Dex is widely used to stimulate browning in adipocytes, we also asked if Ros plays a role in commitment or in stimulating the brown and brite adipose phenotypes. Our results suggested that Mix/Dex or St/Dex induced commitment to differentiation. But the cells did not differentiate into adipocytes, as determined both genotypically and morphologically unless we added Ros to the medium. Ros alone, as a well‐known agonist of PPARγ, does not induce commitment, but it promotes the differentiation of the committed cells into the new adipose phenotype. It is poorly known if the action of Ros is exclusively related to PPARγ activity or to other yet‐unknown activities. In the 3T3‐L1 and F442A, the white adipose clones, Ros enhances adipose differentiation, but it is not required to activate the full lipogenic program, since these cells seem to have an endogenous agonist of PPARγ [79, 80]. It is possible that the brown and brite fat clones have a low concentration, or do not have such intracellular agonists, and therefore Ros must be added to the culture medium to achieve full adipocyte differentiation. Indeed, Ros might have additional roles in brown and brite fat differentiation that are not yet known. We can conclude that Mix/Dex and St/Dex induce commitment to differentiate, whereas Ros promotes the differentiated brown and brite adipose phenotypes.

We showed that St/Dex rapidly induced, within 4 h exposure, the F442A cells to further differentiate to adipocytes [52, 64]. We proposed that St induced two phases for differentiation before clonal expansion in the F442A cells. In the first stage of 4 h induction, St induced progenitor cells to initiate differentiation, and in the second phase of 44 h stabilization, the cells continued into differentiation without the inducer. After these two stages, the cells entered clonal expansion and expressed the adipose phenotype. Before the stabilization stage ends at the time of clonal amplification, it can still be manipulated with various substances to reverse commitment, restoring the cells to their preinduction state [52, 64].

Induction with Mix/Dex and St/Dex of EB5 and EB7 for only 2 and 24 h, respectively, was enough for the cells to undergo full commitment. Our results with the EB5 and EB7 cells suggest that these similarities with the F442A cells hint that the brown and brite preadipocytes might go through similar intracellular molecular events of induction and stabilization before clonal expansion. We can hypothesize that, after induction, stabilization will regulate the progression into differentiation and the generation of new adipocytes. An example is the addition of Ros after induction, i.e., the stabilization stage, stimulates the EB5 and EB7 clones to continue to full expression of the new adipose phenotype. It is not surprising that St is an inducer of cell commitment to differentiation. Besides our results that St induces commitment of F442A, EB5 and EB7, as we showed, and 3T3‐L1 (unpublished results), it has been reported that stauprimide, an analog of St, induces differentiation of embryonic stem cells into the endoderm lineage [81]. All these results suggested that St, and some of its analogs, could induce differentiation in cell progenitors in various tissues or differentiation pathways.

### Induction of adipogenesis depends on GSK3β activity

St is a serine/threonine kinase inhibitor that activates GSK3β [65], so inhibiting this kinase with lithium chloride or with the selective blocker SB415286 inhibited adipose differentiation of F442A cells [64]. Our experiments with the GSK3β selective blocker SB415286 showed that *Pparg2* expression was inhibited in EB5 and EB7 cells more when induced with SDR (about 90%) than with RMD (about 50%). The results suggested that GSK3β is also a crucial kinase in the brown and brite adipogenic pathways. However, since GSK3β inhibition was lower when the cells were induced with RMD than with SDR, it would suggest that commitment with RMD might take place through additional signaling pathways that might not involve the activity of the kinase. Since both cell lines are clones, it is conceivable that the brown and brite adipogenic progenitors might undergo commitment through distinct regulatory steps, yet poorly known. These regulatory pathways would drive the cells to reach a common step, which could be the activation of the PRDM16‐C/EBPβ complex and the canonical transcriptional cascade gene expression [2, 18, 82].

Brown and brite adipogenesis might share some events with white adipogenesis to commit the progenitors into differentiation: (a) A signaling pathway mediated by GSK3β activation that acts as a molecular switch for the differentiation of mesenchymal precursors into adipocytes. This pathway would link GSK3β activity, phosphorylating, directly or indirectly, Thr^179^ and Ser^184^ of C/EBPβ activating its DNA‐binding function [64, 83, 84] to promote *Pparg2* and *Cebpa* transcription [85, 86, 87]. (b) Other signaling pathways, yet unknown, circumventing GSK3β activity while leading to C/EBPβ activation and the expression of *Pparg2*, *Cebpa*, and *Srebf1c* as regulators of brown and brite lipogenic gene expression.

We can propose that the EB5 and EB7 clones are cell progenitors at a very early preadipocyte stage, but are not committed until GSK3β or another signaling pathway is activated. Hence, it appears that Mix/Dex or St/Dex involves the induction of early genes and molecular events that occur after *Prdm16* expression but before *Cebpb*. In contrast, inhibiting GSK3β activity in β‐adrenergically stimulated brown mature adipocytes or cold‐exposed mice increased the thermogenic genes *Fgf21*, *Ucp1*, *Dio2*, and *Ppargc1a* expression [75]. Therefore, GSK3β, a multifunctional kinase involved in many cellular functions [88], seems to play two distinct roles in brown and brite fat differentiation. GSK3β activity is crucial for the induction of adipogenesis in brown, brite, and white preadipocytes, but in contrast, it must be inhibited for β‐adrenergic or cold stimulation of the thermogenic program in brown adipocytes, and not yet explored in brite adipocytes.

*Srebf1a*, the gene encoding another crucial transcription factor in white adipogenesis, SREBP1a [51], was expressed early in the brite cells, similarly to F442A preadipocytes, but less in the brown preadipocytes. *Srebf1a* knockdown during adipogenesis of the human SGBS preadipocyte cell strain, decreased the cell proliferation rate without inducing DNA damage or cell senescence, and without changing lipogenic gene expression. The PCR array of genes involved in cell cycle progression showed that SREBP1a could modulate 12 cell cycle‐related genes [89]. It is conceivable that SREBP1a might have a role in the clonal amplification of the committed cells. Hence, it is tempting to speculate that SREBP1a might regulate the size of the fat lobules in the three adipose tissues, but limiting more the mass growth of the brown adipose organ. Another possible implication is that since *Srebf1a* is highly expressed in the early adipogenesis of the F442A and the EB7 cells, SREBP1a might influence WAT hyperplasia and mass size in obesity.

### Lipid metabolism in EB5 and EB7 clones

Fatty acid availability for thermogenesis and beta‐oxidation is essential for UCP1 proton transport activity in BAT and BRT. Indeed, for the EB5 and EB7 adipocytes to be active in thermogenesis and energy expenditure, the expression of genes involved in metabolic pathways providing fatty acids, such as the lipogenic, the lipolytic, and the glucose and fatty acid transport pathways, is crucial. Lipoprotein lipase serves as a key enzyme in white adipose tissue, catalyzing the hydrolysis of triglycerides in the bloodstream into glycerol and fatty acids at the plasma membrane. This crucial reaction enables the fatty acids' entry into the cytoplasm, where they are used for intracellular triglyceride synthesis and storage. The essential function of LPL in the catabolism of circulating triglycerides is mirrored in the observed increase in *Lpl* expression across all three studied adipocyte types. This induction suggests that the brown EB5 and brite EB7 adipocytes have a pronounced ability to process bloodstream triglycerides, rendering fatty acids available for uptake and likely destined for mitochondrial import and subsequent energy dissipation.

Short‐term cold exposure of mice promotes triglyceride plasma clearance by increasing the breakdown of LPL‐mediated triglyceride‐rich lipoproteins in the bloodstream and promoting fatty acid transport by CD36 into BAT [90]. While *Fabp4* expression increased during adipogenesis of all three cell lines, *Cd36* expression increased only in EB5 and EB7 adipocytes, but not in F442A adipocytes relative to preadipocytes. These data would suggest that *Cd36* might have the function of also promoting fatty acid transport in the EB5 and EB7 adipocytes linked to fatty acid uptake into mitochondria. Similarly, to the expression of *Cd36*, expression of *Cpt1b* showed a substantial increase in EB5 and EB7 adipocyte, whereas this upregulation was not observed in the F442A white adipocytes. CPT1b is the rate‐limiting enzyme in the carnitine shuttle for transporting fatty acids into the mitochondria for beta‐oxidation. It is a regulatory enzyme whose activity is closely linked to the enzyme CPT2, located at the inner mitochondrial membrane that translocates fatty acids from CPT1 into the mitochondrial matrix. BAT shows the highest CPT1 activity among other tissues examined in rats, liver, kidney, heart, white fat, and muscle [91], but its expression was unknown in BRT. Our results also showed that *Cpt1b* expression is indeed significant in brite fat. On the other hand, acetyl‐CoA Carboxylases (ACCs) catalyze the formation of malonyl‐CoA by carboxylation of Acetyl‐CoA and malonyl‐CoA is an important inhibitor of CPT1 activity. There are two isoforms in mammals: ACC1 and ACC2. Fasn is found in the cytosol and is involved in the synthesis of fatty acids, while ACC2 (gene *Acacb*) is found in the outer mitochondrial membrane and catalyzes the formation of malonyl‐CoA (reviewed in Wang *et al*. [92]). The increase in *Acacb* expression in the adipocytes in comparison with their corresponding preadipocytes was about 500‐fold for EB5, 1200‐fold for EB7, while only 50‐fold for F442A. It is possible that ACC2 activity could be crucial in brown and brite fat to regulate the thermogenic pathway by modulating the concentration of malonyl‐CoA, an inhibitor of CPT1b activity. For example, downregulation of *Acacb* would lead to a lower concentration of malonyl‐CoA, preventing CPT1b inhibition to drive thermogenesis. Taken together, our results suggest that the coordination of the activities of CD36, CPT1b, and ACC2 is crucial to partition the fatty acids into mitochondria for thermogenesis in brown fat and remarkably in brite fat.

Stearoyl CoA desaturase‐1 (Scd1) is a rate‐limiting enzyme that catalyzes the biosynthesis of MUFA, palmitoleyl‐CoA and oleyl‐CoA from palmitoyl‐CoA and stearoyl‐CoA, respectively. Our analysis revealed a substantial upregulation of *Scd1* expression in EB5 and EB7 adipocytes compared to their respective preadipocytes, with the extent of this upregulation being notably more pronounced than in the F442A clone. These data suggest that Scd1 must have a key function in BAT and BRT metabolism by providing these adipocytes with palmitoleic and oleic fatty acids. Dietary oleate partially compensated for hypothermia and hypoglycemia in the cold‐exposed *Scd1* ^−/−^ mice, suggesting an important role of MUFAs in thermoregulation [93]. It is compelling to suggest that finding ways to stimulate the activity of Scd1 in BAT or BRT might promote thermogenesis and energy expenditure by providing MUFAs for beta‐oxidation by UCP1. Indeed, the impaired temperature control in cold‐exposed *Scd1*‐deficient mice agrees with preserving or stimulating the activity of *Scd1* to drive thermogenesis. This is consistent with the observation that Scd1 promotes adipose lipid mobilization in response to cold stimulation [94]. Experiments silencing the *Scd1* gene in the EB5 and EB7 adipocytes might show if *Scd1* deficiency might lead to a downregulation of *Ucp1* expression and thermogenesis in brown and brite adipocytes, and hence to assign a crucial role of Scd1 to reroute the concentration of saturated fatty acids to MUFAs and the use of these for thermogenesis.

Since both EB5 and EB7 adipocytes express genes essential for various aspects of carbohydrate and lipid metabolism, it is important to determine their physiological competence. The β_3_‐adrenergic response demonstrates the physiological capabilities of brown adipocytes, as indicated by lipolysis, UCP1 content, and oxygen consumption (refer to Introduction). We found that norepinephrine, a selective β_3_‐AR agonist, increased UCP1 protein levels and lipolytic activity in EB5 brown and EB7 beige adipocytes, confirming their functional metabolic competence. Interestingly, the EB7 adipocytes, which are brite/beige adipocytes, also exhibited the selective β_3_‐adrenergic response, suggesting that brite/beige adipose tissue likely responds to β_3_‐adrenergic stimulation.

## Conclusion

The EB5 and EB7 murine embryonic clonal cells differentiate into brown and brite adipocytes and express their corresponding tissue‐specific gene markers. Interestingly, we also described the crucial role of GSK3β, a molecular switch in various differentiation processes, to regulate brown and brite fat differentiation. Additionally, the differentiated adipocytes express the crucial genes of the carbohydrate, lipid, and thermogenic pathways of brown and brite fat. Studies using EB5 and EB7 should complement other models for exploring adipocyte biology. First, given that the cells are derived from 11 to 12 embryos, they may aid to better understand commitment, differentiation, and/or development of brown and beige cells. Second, these cell lines may be used to study crucial enzymes involved in lipid and carbohydrate metabolism and discern how different adipocyte populations make different contributions to metabolism. Third, these cells may be useful to identify and/or characterize drug candidates targeting energy expenditure for treating obesity and related metabolic complications. In sum, the ability to perform large‐scale cultivation with clonally pure EB5 and EB7 cells should aid in studies‐related developmental biology, intermediary metabolism, and/or drug discovery.

## Conflict of interest

The authors declare they own stock in a start‐up biotech company. CV‐V is a consultant. WK‐H is a founder and serves on the board without pay. These relationships have been disclosed and have not influenced the content of this article.

## Author contributions

WK‐H and CV‐V designed the experiments. CV‐V, MM‐M, CPH‐M, LIC‐R, and AV‐S performed the experiments. CV‐V and WK‐H analyzed the data. CV‐V and WK‐H wrote the article. All authors read and approved the final article.

## Supporting information


**Table S1.** Names, and acronyms of proteins and genes accordingly to the NCBI GenBank and GenPept databases.

## Data Availability

The authors confirm that the data supporting the findings of this study are available within the article and its supplementary materials.
