# Supplementary material for: Gene expression and characterization of clonally derived murine embryonic brown and brite adipocytes
Source: FEBS Open Bio. 2024 Jul 7;14(9):1503–25. doi: 10.1002/2211-5463.13861 (PMC11492321; doi:10.1002/2211-5463.13861)
Supplement: Supplementary file 1 — Table S1. Names, and acronyms of proteins and genes accordingly to the NCBI GenBank and GenPept databases. [file FEB4-14-1503-s001.docx]

**Table S1. Names, and acronyms of proteins and genes accordingly to the NCBI GenBank and GenPept databases.**

| **Protein** | **GenPept Accession Number** | **Gene name(s)** | | **GenBank Accession Number** | **Full Name(s)** |
| --- | --- | --- | --- | --- | --- |
| ACC2 | NP_598665 | *Acacb* | | NM_133904 | Acetyl-CoA carboxylase 2; ACC-beta. |
| Adiponectin | NP_033735 | *Adipoq* | | NM_009605 | Adiponectin; C1Q and collagen domain containing. |
| Atgl | NP_080078 | *Atgl; Pnpla2* | | NM_025802 | Patatin-like phospholipase domain-containing protein 2 (Pnpla2);Adipose Triglyceride Lipase; Desnutrin. |
| CD36 | NP_001153030 | | *Cd36; FAT; GPIV; Scarb3* | NM_001159558 | CD36 molecule (Cd36); platelet glycoprotein 4; PAS-4; GPIIIB; PAS IV; glycoprotein IIIb; platelet glycoprotein IV; fatty acid translocase. |
| C/EBP α | NP_031704 | | *Cebpa* | NM_007678 | CCAAT/enhancer binding protein (C/EBP) alpha |
| C/EBP β | NP_034013 | | *Cebpb* | NM_009883 | CCAAT/enhancer binding protein (C/EBP) beta ; AGP/EBP; interleukin-6-dependent-binding protein; liver-enriched transcriptional activator; nuclear protein Il6; C/EBP BETA |
| CIDE-A | NP_031728 | *Cidea* | | NM_007702 | Cell death activator CIDE-A; cell death-inducing DFFA-like effector A |
| CPT1b | NP_034078 | *Cpt1b; Cpt1;*  *Cpt1-m; Cpti;*  *Cpti-m;*  *M-cpti* | | NM_009948 | Carnitine O-palmitoyltransferase 1b (CPT1b); muscle-type carnitine palmitoyltransferase I; carnitine O-palmitoyltransferase 1 |
| FABP4 | NP_077717 | *Fabp4* | | NM_024406 | Fatty acid binding protein 4, adipocyte (FABP4); protein 422; P2 adipocyte protein; myelin P2 protein homolog; 3T3-L1 lipid-binding protein; adipocyte lipid-binding protein; adipocyte-type fatty acid-binding protein; adipocyte protein aP2 |
| Fasn | NP_032014 | *Fasn* | | NM_007988 | Fatty acid synthase (Fasn); type I Fatty Acid Synthase; A630082H08Rik; FAS |
| GPD1 | NP_034401 | *Gpd1; Gdc-1; Gdc1; GPD-C; GPDH-C* | | NM_010271 | Glycerol-3-phosphate dehydrogenase 1 (soluble) (Gpd1); glycerol-3-phosphate dehydrogenase [NAD(+)],cytoplasmic; glycerolphosphate dehydrogenase 1, cytoplasmic adult |
| Leptin | NP_032519 | *Lep* | | NM_008493 | Leptin precursor; obesity factor |
| Lhx8 | NP_034843 | *Lhx8; L3; Lhx7* | | NM_010713 | LIM homeobox protein 8 (Lhx8); LIM homeobox protein 7; LIM/homeobox protein Lhx7; LIM homeo box protein 8 |
| Lipe | NP_034849 | *Lipe* | | NM_010719 | Hormone sensitive lipase (Lipe); hormone-sensitive lipase; retinyl ester hydrolase; monoacylglycerol lipase HSL |
| LPL | NP_032535 | *Lpl* | | NM_008509 | Lipoprotein lipase (LPL); Phospholipase A1 |
| ChREBP | NP_067430 | *Mlxipl* | | NM_021455 | MLX interacting protein-like (Mlxipl); putative hepatic transcription factor; MLX interactor; Williams-Beuren syndrome chromosome region 14 homolog; Max-like protein; carbohydrate-responsive element-binding protein; MLX interacting protein-like beta; carbohydrate-responsive element-binding protein |
| Myf-5 | NP_032682 | *Myf5* | | NM_008656 | Myogenic factor 5 (Myf5; myogenic regulatory factor MYF-5 |
| Pgc-1α | NP_032930 | *Ppargc1a* | | NM_008904 | peroxisome proliferative activated receptor, gamma, coactivator 1 alpha (Ppargc1a); PPAR-gamma coactivator 1-alpha |
| PPARγ | NP_032930 | *Pparg2; Nr1c3; PPAR-gamma; PPAR-gamma2; PPARgamma* | | NM_011146 | Peroxisome proliferator activated receptor gamma (Pparg); peroxisome proliferator activated receptor gamma 2; peroxisome proliferator activated receptor gamma 4; nuclear receptor subfamily 1 group C member 3 |
| PRDM16 | NP_081780 | *Prdm16; 5730557K01Rik; csp1; mel1* | | NM_027504 | PR domain containing 16 (Prdm16); transcription factor MEL1; MDS1/EVI1-like gene 1; PR domain-containing protein 16; PR domain zinc finger protein 16; line 27; histone-lysine N-methyltransferase PRDM16 |
| Rplp0 | NP_031501 | *Rplp0; 36B4; Arbp; L10E* | | NM_007475 | Ribosomal protein, large, P0 (Rplp0); acidic ribosomal phosphoprotein P0; 60S ribosomal protein L10E; 60S acidic ribosomal protein P0 |
| Scd1 | NP_033153 | *Scd1* | | NM_009127 | Stearoyl-Coenzyme A desaturase 1 (Scd1); delta(9)-desaturase 1; fatty acid desaturase 1; stearoyl-CoA desaturase 1; asebia; acyl-CoA desaturase 1 |
| Glut4 | NP_033230 | *Slc2a4; Glut-4; Glut4; GT2* | | NM_009204 | Solute carrier family 2 (facilitated glucose transporter), member 4 (Slc2a4) glucose transporter type 4, insulin-responsive. |
| SREBP1a | NP_035610 | *Srebf1a* | | NM_011480 | Sterol regulatory element-binding protein 1 isoform a (SREBP1a); adipocyte determination- and differentiation-dependent ADD1; bHLHd1; sterol regulatory element-binding protein 1a. |
| SREBP1c | NP_001345243 | *Srebf1c* | | NM_001358314 | Sterol regulatory element-binding protein 1 isoform c (SREBP1c). |
| Tcf21 | NP_035675 | *Tcf21; bHLHa23; capsulin; epc; epicardin; Pod-1;Pod1* | | NM_011545 | Transcription factor 21 (Tcf21); podocyte-expressed 1. |
| Tmem26 | NP_808462 | *Tmem26* | | NM_177794 | Transmembrane protein 26 |
| UCP1 | NP_033489 | *Ucp1* | | NM_009463 | uncoupling protein 1 (mitochondrial, proton carrier) (Ucp1); mitochondrial brown fat uncoupling protein 1; UCP 1; thermogenin; solute carrier family 25 member 7. |
| Zic1 | NP_033599 | *Zic1; ZNF201* | | NM_009573 | Zinc finger protein of the cerebellum 1 (Zic1); zinc finger protein ZIC 1; Zic family member 1. |
